# *A Case of Disease Produced by Taking One Ounce of Corrosive Sublimate, Successfully Treated. Communicated to* Dr. Coxe

**Published:** 1807-10

**Authors:** Wm. Budd


### *A Case of Disease produced by taking one Ounce of Corrosive Sublimate, successfully treated. Communicated to* Dr. Coxe, *by* Dr. Wm. Budd.

DECEMBER 11, 1799, I received a note, requesting my attendance on a young lady, about 12 o'clock, who was represented to be very ill. I hasted there, and found her walking about the room without much appearance of extreme danger. On conversing with her concerning the subject of her complaint, I found, that twenty-four hours previous to my being called, she had been suddenly seized with violent pains in the stomach, which were directly followed by profuse vomiting of blood, which symptoms still continued at intervals: the abdomen was swelled as large as a woman's in her seventh month of pregnancy, was very hard, and she had pains at regular intervals, similar to pains in labour; she complained of great thirst, but was not able to retain what she drank any length of time; pulse rather more frequent than usual, but not full: the above symptoms gave me reason to suspect they were owing to some irregularity in the usual periodical discharges, or perhaps she might be pregnant, and the violent pains caused by that. I made further inquiry, in hopes that she would relate some circumstance that would unfold the real cause of her disease, but all to no purpose, for she declared she had not taken any thing that could have produced that effect, and that she had been regular but two weeks before, and experienced good health previously to that period. I directed about twelve ounces of blood to be taken from the arm, as soon as it could be done conveniently, and gave her two grains of opium in pills, hoping she would be eased by them, but they were almost instantly thrown up. 12th. The swelling still continued, attended with great pain in the stomach and bowels; very thirsty, and the bowels have not been freely opened since yesterday; the bleeding of last night produced no relief; I directed the same quantity to be taken again, and to give a table spoonful of the ol. ricini every hour, until the bowels should be freely opened.

13th. The swelling of the abdomen has suddenly disappeared during the last night, and the pain likewise abated, so that the patient got some rest; but this morning the swelling returned with the usual symptoms of pain, and great heat or burning of the stomach, and occasional vomiting or belching of blood; for, as she lay on her back,

 she she would raise considerable quantities of blood, mixed with water, by a peculiar action of the stomach, without any exertion on her part; the stomach has become so very irritable that she could with difficulty bear to be touched with the hand, or even to sustain the weight of the bed clothes. She now complains of soreness in the throat and fauces, which partly confirms me in what I frequently suspected, that she had taken some poisonous medicine, and that is most likely to be a mercurial poison, from the affection of the mouth and throat.

I ordered the oil to be frequently repeated through the day, with a double view; 1 That it would be most likely to preserve the stomach and bowels from injury, and from any corrosive matter that might be contained in them; and, 2. The good effects that would be derived from its purgative quality: I likewise directed about ten ounces of blood to be taken, and a large blister to be applied to the region of the stomach, as well as one to each wrist, also her drinks to be of the most diluting kind, such as flax-seed tea, barley water, &c.

14th. I found her generally relieved from the pains in the stomach and bowels; the oil operated very well, and the blisters drew considerably; she says the pain is chiefly removed from the stomach, but has a pricking sensation, as she describes, like sword points sticking in it; troubled with fainting fits. This afternoon her pains returned with redoubled violence, and are attended with considerable fever; the affection of the head still continues, as well as the discharge of blood from the stomach. She now thought proper to inform me, through the medium of a friend, that she had taken on the day before I was called, twenty-five cents worth of merc. cor. sub. which from the general price of that medicine, must amount to an ounce or four hundred and eighty grains; her situation now appeared to be desperate; indeed it is strange, that instant death did not ensue from the enormous dose of poison; the pricking pain, as above described, proceeded, as I suppose, from some of the sublimate in its native state in the stomach; I determined therefore to try the effects of a strong solution of vegetable alkali, which would cause a speedy decomposition, and render the sublimate inoffensive; the alkaline solution answered my expectation, for it produced instant relief, and remained longer in the stomach than any liquid she had taken before.

15th. Continues better; ordered the solution and oil to be repeated.

16th,

16th. Swelling has increased very much since yesterday: complains of great pain in the stomach and bowels, throat very sore, and has frequent fits; pulse full and hard. Catamenia made their appearance during last night, and increase to an alarming degree; ordered her to be bled, and take ten grains of alum, with about an equal quantity of sal nitri every two hours till she should get relief, and cold vinegar cloths to be applied to the pubes.

17th. Mœnorrhagia abated, stomach retains its medicine, but is still very sensible to the touch; bowels freely opened; drinks flax-seed tea, and is able to take a small quantity of light nourishment. Ordered small quantities of sweet oil at intervals through the day to be taken, and if the bowels should not be freely opened, to have an injection.

18th. Continues to mend, and takes the oil two or three times a day.

20th. The abdomen frequently swells and becomes very hard, but disappears by the operation of the medicine.

January 21, 1800. I was again sent for to see my old patient, who is afflicted in the manner before described, with an additional swelling in the epigastric region of the left side, and a sense of weight and hardness, which she says she has felt for several days; the stomach still remains very irritable and frequently rejects what is taken in it. I resumed my former mode of practice, which appeared to me to be the best, and perhaps only means by which I could effect a cure.

22d. I found her relieved from most of her complaints, in consequeuce, I suppose, of a copious discharge of matter from the stomach, which resembled pus, and which she says had a saltish taste: I directed her to take a moderate portion of nourishing diet, and to use oil, whenever the bowels required opening.

29th. She still continues mending, and frequently vomits the aforementioned matter, which vomiting is preceded by a sense of pain and weight in the stomach.

*N. B.* I heard from her some time after: she was in good health, except some slight affection of the stomach, which perhaps may be increased by a strong propensity to eat Cayenne pepper since her illness.

*Philadelphia, August* 1, 1805.

